# Evolution of the HIV-1 *nef *gene in HLA-B*57 Positive Elite Suppressors

**DOI:** 10.1186/1742-4690-7-94

**Published:** 2010-11-08

**Authors:** Maria Salgado, Timothy P Brennan, Karen A O'Connell, Justin R Bailey, Stuart C Ray, Robert F Siliciano, Joel N Blankson

**Affiliations:** 1 Department of Medicine, Johns Hopkins University School of Medicine. 733 N. Broadway, Baltimore MD 21205, USA; 2 Howard Hughes Medical Institute. Johns Hopkins University School of Medicine. 733 N. Broadway, Baltimore MD 21205, USA

## Abstract

Elite controllers or suppressors (ES) are HIV-1 infected patients who maintain viral loads of < 50 copies/ml without antiretroviral therapy. CD8+ T cells are thought to play a key role in the control of viral replication and exert selective pressure on *gag *and *nef *in HLA-B*57 positive ES. We previously showed evolution in the *gag *gene of ES which surprisingly was mostly due to synonymous mutations rather than non-synonymous mutation in targeted CTL epitopes. This finding could be the result of structural constraints on Gag, and we therefore examined the less conserved *nef *gene. We found slow evolution of *nef *in plasma virus in some ES. This evolution is mostly due to synonymous mutations and occurs at a rate similar to that seen in the *gag *gene in the same patients. The results provide further evidence of ongoing viral replication in ES and suggest that the *nef *and *gag *genes in these patients respond similarly to selective pressure from the host.

## Findings

The mechanisms responsible for the control of the HIV-1 replication in elite suppressors are not fully understood [1-3]. Replication competent virus has been isolated from some ES [4-6] and genotypic [4], phenotypic [4], and epidemiologic [7] analyses have suggested that these isolates are generally fully pathogenic. Thus it appears that in many cases, host factors rather than infection with defective virus are responsible for the elite control of viral replication. The HLA-B*57 allele is overrepresented in ES [8-14] which suggests an important role of CD8+ T cells. These cells have been shown to exert selective pressure on HLA-B*57 restricted epitopes in ES [15-17] and LTNPs [18], and we have previously documented evidence of evolution in the *gag *gene in plasma virus of HLA-B*57 positive ES over a 5 year period [19]. However, some studies have suggested that Gag is preferentially targeted by CD8+ T cells in patients who control viremia [12,20] and it is therefore possible that viral evolution in ES is limited to this gene. To test this hypothesis, we analyzed proviral and plasma *nef *sequences in ES over a 6 year period and compared the rate of evolution in these two compartments to the rate of evolution of observed for the *gag *gene.

Four previously described HLA-B*57 ES patients were studied [16,21]. Viral RNA was isolated from plasma, and genomic DNA was purified from resting CD4+ T cells as described previously [16]. To limit PCR resampling, *nef *genes were amplified from provirus in genomic DNA and from plasma-derived RNA by limiting dilution "digital" nested PCR using previously described primers and conditions [21]. PCR products were directly sequenced using an ABI PRISM 3700 DNA analyzer (Applied Biosystems). Chromatograms were manually examined for the presence of double peaks indicative of two templates per sequencing reaction. Such sequences were discarded. Sequences were assembled using CodonCode Aligner, version 1.3.1, aligned using ClustalX, and the alignments were manually adjusted in Bioedit. Sequences were translated in Bioedit, and the mean number of amino acid differences between all provirus *nef *and all plasma virus *nef *sequences from each patient was calculated.

### Phylogenetic analysis and statistics

All independent clonal sequences obtained were included in the phylogenetic analysis, with the exception of sequences showing APOBEC3G/F-mediated hypermutation, which were removed. Sequences [GenBank: FJ430356 to FJ430471 and HQ448774 to HQ448852] subsequently were handled as previously described [19]. Classical, maximum-likelihood, and Bayesian phylogenetic reconstruction for each patient was performed as previously described [19]. Non-synonymous and synonymous p-distance calculations and the number of differences were based on the Nei-Gojobori method [22] and were calculated by comparing grouped sequences from the early time points in each patient to sequences from the later time points using MEGA 4.0 software [23].

We performed a longitudinal analysis of viral sequences in 4 ES HLA-B*57+ patients in order to determine whether viral evolution occurred in *nef*. A median of 12 independent *nef *clones (range 3 to 17) were amplified from plasma virus of each of the four ES. In three patients, we also amplified a median of 9 *nef *sequences from proviral DNA in resting CD4+ T cells (range 6 to 16). These new sequences were compared to sequences obtained from the patients 5 to 6 years earlier.

**Figure 1:**
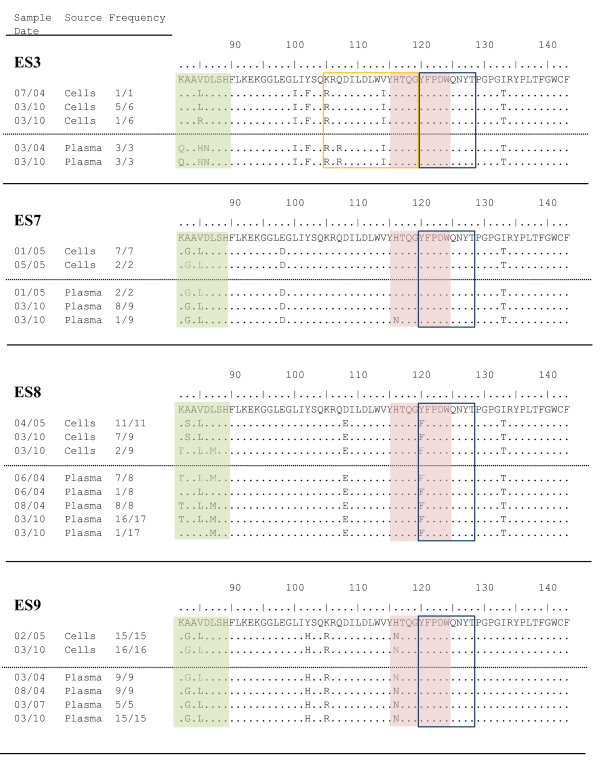
**Relevant sequence regions from clonal, *nef *amplified from plasma or from resting CD4+T cells**. The date of sample acquisition and number of clones that are identical to the displayed sequences are noted. The HLA-B*57-restricted epitope YT9 (Nef 120-128) and the undefined epitope KG15 (nef 105-119) in ES3 are denoted by empty boxes outlined in blue and yellow respectively. The HLA-B*57-restricted epitopes KF9 (Nef 82-90), and HW9 (Nef 116-124), are denoted by boxes shaded in green and pink respectively. Sequences from 2004 and 2005 for ES3, ES7, ES8, and ES9 have been previously reported and are shown for comparative purposes only.

A marked discordance between plasma and proviral sequences was seen at the HLA-B*57 restricted epitope KF9 (Nef 82-90) in ES3 and ES8 (Figure 1). In both patients, the majority of the plasma clones were different at 2 or 3 amino acids from the majority of the proviral clones. There was no evidence of discordance in this epitope in ES7 and ES9 or at the other 2 HLA-B*57 restricted epitopes in any patient, but we continued to see a previously described escape mutation (Q107R) in an undefined epitope in plasma clones in ES3 (KG15, Nef 105-119) [21]. This mutation was not seen in proviral clones. While there was discordance between plasma and proviral clones at both early and late time points, there did not appear to be significant evolution in any of the epitopes in either compartment over a 6 year period.

**Figure 2:**
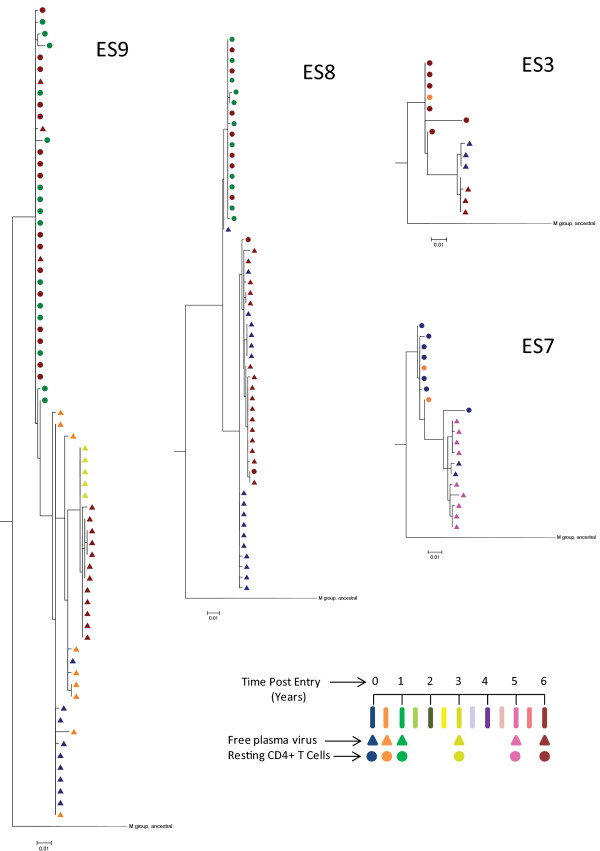
**Phylogenetic analysis of *nef *in the 4 elite suppressor patients**. Phylogenies were estimated by using a "classical" approach, functioning under maximum-likeluhood (ML) optimality criterion. All sequences are clonal, and APOBEC-mediated hypermutated sequences were removed from analysis. Colors indicate time, with the scale below in years. Triangles represent clonal plasma sequences, circles represent proviral sequences from CD4+ resting T cells.

In order to determine whether evolution was occurring in other regions of the gene, phylogenetic analyses were performed. As shown in Figure 2, there was a striking segregation of plasma and proviral *nef *sequences for all 4 patients. Proviral sequences from all time points were clustered together and were clearly ancestral to plasma clones from all time points. Furthermore, in ES9 and to a lesser extent ES8, some or all of the plasma clones obtained in 2004 or 2005 (year 0 and 1 respectively) were ancestral to those obtained in 2007 (year 3) and 2010 (year 6). A clear pattern was not seen for ES3 and ES7, possibly due to the lower number of clones available from these patients. The fact that proviral clones were generally ancestral to plasma clones is consistent with the model that proviral clones represent archived HIV-1 in latent reservoirs. As with *gag *[19], there is little re-seeding of the latent reservoir by plasma *nef *clones in ES.

**Figure 3:**
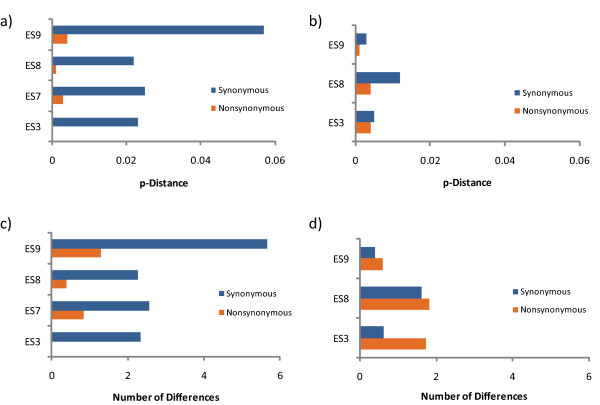
**Analysis of synonymous and non-synonymous mutation in the plasma virus and proviral compartments**. Shown are p-distance values for plasma (A) and proviral (B) sequences as determined by comparing early and late samples for each patient utilizing the Nei-Gojobori method. The numbers of differences also were calculated for plasma (C) and proviral (D) sequences using the Nei-Gojobori method.

In order to further elucidate the observed evolution seen in plasma clones in some ES, we used the Nei-Gojobori method to compare the p-distances of non-synonymous and synonymous mutations between early and late plasma and proviral sequences (Figure 3). This parameter normalizes the frequency of substitutions to the number of possible synonymous or non-synonymous sites, permitting comparisons between the two. Figure 3A shows that synonymous mutations were a more significant factor in the divergence of plasma virus than were non-synonymous mutations in all 4 patients. The same finding was seen with proviral sequences (Figure 3B). We also calculated the number of differences between early and late plasma and proviral sequences in these patients (Figure 3C and 3D). Very few changes were seen in proviral sequences and there were comparable levels of synonymous and non-synonymous mutations. In some cases this may have been partially due to a low level of plasma virus entering the latent reservoir. In contrast, there were more changes seen in plasma virus and these were mostly synonymous mutations. Taken together, these data illustrate that overall there is significantly more synonymous mutation than non-synonymous mutation and that mutations are more frequent in the plasma virus than in the provirus.

**Figure 4:**
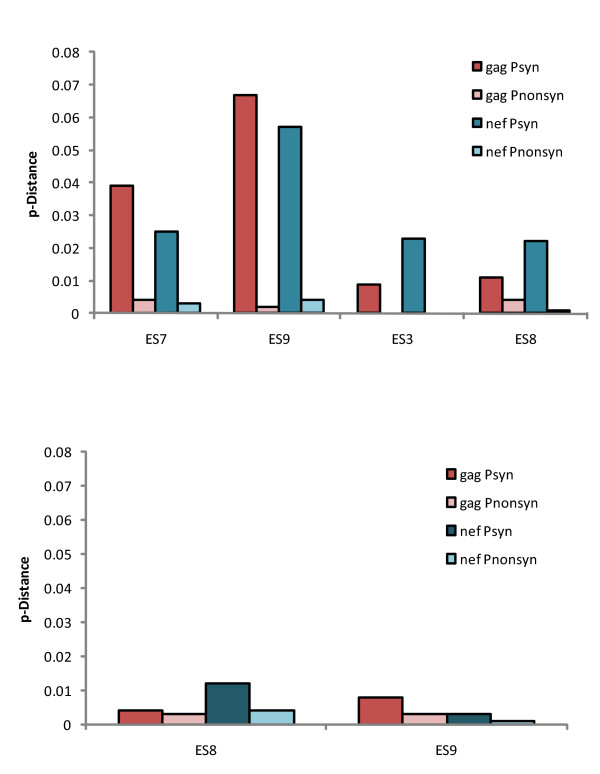
**Comparison of synonymous and non-synonymous mutations in *nef *and *gag *for plasma (top panel) and proviral (bottom panel) clones**. P-distance values were calculated for each patient utilizing the Nei-Gojobori method.

We have previously characterized CD8+ T cell responses to Gag [16] and Nef [21] for all 4ES. A median of only 1.5 epitopes in Nef were recognized, the majority of which were HLA-B*57 restricted. A similar pattern was seen in Gag with a median of 3.5 epitopes recognized in this protein, most of which were also HLA-B*57 restricted. To determine whether selective pressure exerted by these T cell responses on these epitopes resulted in comparable levels of evolution, we compared the p-distances for *nef *to those we previously calculated for *gag *(Figure 4). The same pattern of a predominance of synonymous mutations was seen in the two genes with a much higher degree of evolution seen in plasma clones (Figure 4A) than proviral clones (Figure 4B). Furthermore, the p-distances were similar in the *gag *and *nef *genes for all 4ES, suggesting that comparable degrees of evolution have occurred in the two genes.

There is substantial evidence that HIV-specific CD8+ T cells that are ES are functionally superior to those found in patients with progressive disease [13,24-28]. Some studies suggest that Gag is preferentially targeted in patients who control HIV-1 viremia [12,20] and others have shown that Gag-specific CD8+ T cells are better at inhibiting viral replication than T cells that target Nef [27]. Gag has structural constraints that limit the rate of virologic escape. Also, mutations in some Gag HLA-B*57-restricted epitopes occur at a substantial fitness cost [29,30], leading to reversion to wild type sequence when mutants are transmitted to HLA-B*57 negative individuals [31,32]. In contrast, Nef is less constrained and there is not as much evidence for diminished fitness of escape variants. In fact, in one study, mutations in the Nef KF9 epitope did not revert to wild type following transmission to HLA-B*57 negative patients [33].

We have previously shown that CD8+ T cells exert selective pressure on both Gag [16] and Nef [21] in plasma virus. We also recently documented evolution in the *gag *gene in plasma virus in ES [19]. This evolution was mostly seen in regions outside of epitopes and was due to synonymous mutations. We hypothesized that the lack of non-synonymous mutations was a result of the structural constraints on Gag. One would thus expect to see a higher rate of non-synonymous mutations in Nef. However, in all four patients, we saw very few non-synonymous mutations and a comparable frequency of synonymous mutations in both genes. This analysis is technically challenging because it involves the amplification of multiple clonal sequences from plasma and resting CD4+ T cells of patients who often maintain viral loads of < 1 copy/ml [13,34-36]. The results suggest that in both Gag and Nef, after early virologic escape occurs, there is little continuing evolution in epitopes as a balance between immune evasion and viral fitness is achieved. However, non-synonymous mutations accumulate in both genes in plasma virus as a result of low level ongoing replication. This finding may have implications for the design of HIV-1 vaccines.

## Competing interests

The authors declare that they have no competing interests.

## Authors' contributions

MS carried out the molecular genetic studies, participated in the sequence alignment and drafted the manuscript. TPB performed sequence analysis. KAO and JRB carried out the molecular studies. SCR performed sequence analysis and participated in study design. RFS participated in study design and helped to draft the manuscript JNB conceived of the study, and participated in its design and coordination and helped to draft the manuscript. All authors read and approved the final manuscript.
